# Peri- and Postoperative Outcomes for Obstructive Sleep Apnoea Patients after Bariatric Surgery—a Systematic Review and Meta-analysis

**DOI:** 10.1007/s11695-023-06557-8

**Published:** 2023-05-04

**Authors:** Tarun Katasani, Guy Holt, Waleed Al-Khyatt, Iskandar Idris

**Affiliations:** 1 Medical School, University of Nottingham, Nottingham, UK; 2 Royal Derby Hospital, East Midlands Bariatric & Metabolic Institute, Derby, DE22 3NE UK; 3 Clinical, Metabolic and Molecular Physiology Research Group, MRC-Versus Arthritis Centre for Musculoskeletal Ageing Research, Royal Derby Hospital Centre, University of Nottingham, Derby, UK; 4 National Institute for Health Research (NIHR) Nottingham Biomedical Research Centre, Nottingham, UK

**Keywords:** Sleep apnoea, Bariatric surgery, Complications, Length of stay, Cardiovascular, Continuous positive airway pressure, CPAP

## Abstract

**Background:**

Obstructive sleep apnoea (OSA) is prevalent among patients undergoing bariatric surgery. Previous studies have reported a higher risk of complications, ICU admission and longer length of stay in patients with OSA following surgery. However, clinical outcomes following bariatric surgery are unclear. The hypothesis is that patients with OSA will have an increased risk of these outcome measures after bariatric surgery.

**Methods:**

A systematic review and meta-analysis were performed to answer the research question. Searches for bariatric surgery and obstructive sleep apnoea were performed using PubMed and Ovid Medline. Studies which compared OSA and non-OSA patients undergoing bariatric surgery and used outcome measures that included length of stay, risk of complications, 30-day readmission and need for ICU admission were selected for the systematic review. Comparable datasets from these studies were used for the meta-analysis.

**Results:**

Patients with OSA are at greater risk of complications after bariatric surgery (RR = 1.23 [CI: 1.01, 1.5], *P* = 0.04), driven mostly by an increased risk of cardiac complications (RR = 2.44 [CI: 1.26, 4.76], *P* = 0.009). There were no significant differences between the OSA and non-OSA cohorts in the other outcome variables (respiratory complications, length of stay, 30-day readmission and need for ICU admission).

**Conclusion:**

Following bariatric surgery, patients with OSA must be managed carefully due to the increased risk of cardiac complications. However, patients with OSA are not more likely to require a longer length of stay or readmission.

**Graphical Abstract:**

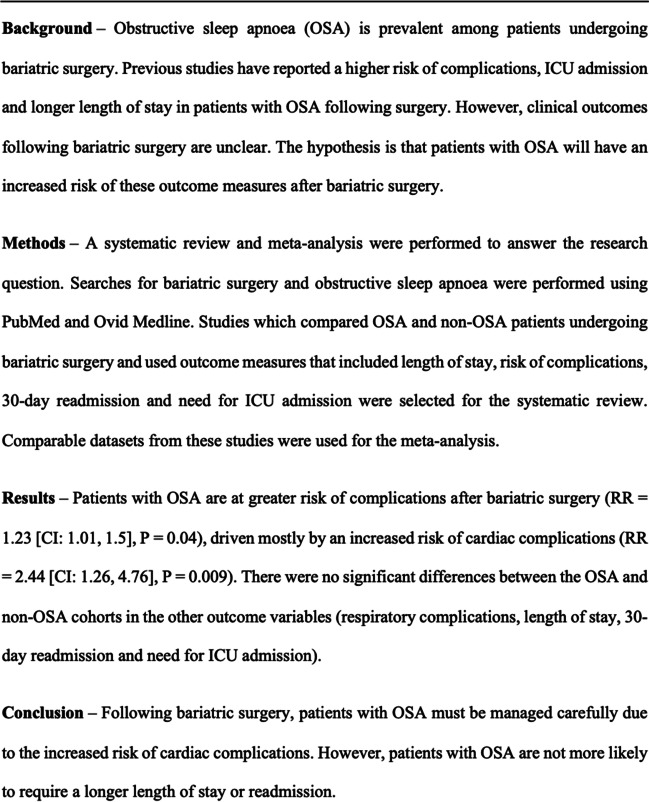

## Introduction

Obstructive sleep apnoea (OSA) is caused by the relaxation of muscles that support the soft tissues in the throat leading to partial or complete closure of the upper airways [1]. Repeated apnoea and hypopnoea during sleep cause intermittent hypoxia, hypercapnia and excess daytime somnolence and are associated with significant cardio-metabolic comorbidity, including cardiovascular mortality [2]. Recent studies have estimated OSA to affect 17% of women and 35% of men [3], higher than previous estimates of around 2% and 4% respectively [4]. OSA is diagnosed based on polysomnography [5] of overnight oximetry to determine apnoea (complete cessation of airflow for 10 s) or hypopnoea (cessation of airflow by about 30% for 10 s) events. The total number of apnoea and hypopnea per total number of hours of sleep is the apnoea Hypopnea index (AHI) and AHI of > 15 indicate moderate OSA and AHI of > 30 is classed as severe OSA. Screening for OSA is often undertaken by use of questionnaires. The Berlin questionnaire is often used in the primary care setting. The STOP-Bang questionnaire was created for preoperative screening. The Epworth sleepiness scale is also used but is less sensitive in detecting OSA in patients [3].

Obesity, defined as having a body mass index (BMI) > 30 kg/m^2^, is the most significant risk factor for OSA. A 10% rise in body weight leads to an approximate 30% increase in the AHI [6]. A high BMI along with excessive daytime sleepiness are often enough to diagnose or suspect OSA in most patients [7]. Additional risk factors for OSA are male gender, family history of OSA syndrome, long-term excessive alcohol intake and long-term smoking [8].

Continuous positive airway pressure (CPAP) therapy is widely used to treat moderate-to-severe OSA [9] by preventing the closure of the upper airways during sleep. It is highly effective, safe to use and significantly improves patients’ quality of sleep [10]. Despite this, adherence to CPAP treatment is poor among patients with OSA. Initial improvements to sleep after starting treatment may cause patients to be less strict with their use of the CPAP machine [11]. In addition to CPAP therapy, increasing evidence has shown that weight loss is associated with significant reductions in the AHI and improvements in the symptoms of OSA [8].

Bariatric surgery is the most effective strategy to reduce and maintain long-term weight loss among patients living with obesity and is associated with significant benefits in the treatment of obesity-related comorbidities such as type 2 diabetes, hypertension, dyslipidaemia and OSA [12]. OSA is prevalent among patients undergoing bariatric surgery [13], but the risks involved with bariatric surgery prevent it from being more widely used as its first-line treatment [12]. Nevertheless, by addressing the major risk factor for OSA, bariatric surgery has shown improvements in the symptoms of OSA [14, 15] but concerns persist regarding the risks of postoperative complications following bariatric surgery [16].

Following general surgery, patients with OSA are at greater risk of complications, stay longer in the hospital and are more likely to require ICU admission than patients without OSA [17]. The use of CPAP machines pre- and postoperatively has greatly reduced these risks in patients with and without OSA [18]. Thus, patients with OSA need to be carefully evaluated before and after bariatric surgery for the presence of OSA and/or compliance with CPAP therapy to reduce their length of hospital stay and risks of complications. The risks of complications among patients with OSA undergoing bariatric surgery however remain unclear. Due to these concerns and ongoing uncertainties regarding the risks of postoperative complications among patients with OSA who undergo bariatric surgery, we undertook a systematic review and meta-analysis to evaluate the outcomes and risks of patients with OSA after bariatric surgery.

## Methods

During the preparation of our manuscript, we strictly followed the recommended reporting items for the Preferred Reporting Items for Systematic Reviews and Meta-Analyses (PRISMA) statement guidelines [19].

The initial search was performed on PubMed and Ovid Medline using the MeSH terms ‘bariatric surgery’ and ‘sleep apnoea, obstructive’. The study design was not specified to increase the sensitivity of the search. Studies had to meet two criteria to be suitable for the systematic review and meta-analysis. Firstly, the intervention group must be patients with OSA and the control group must be patients without OSA. Secondly, the outcome measures must include the length of hospital stay, risk of complications, ICU admission or readmission within 30 days. Only randomised controlled trials (RCTs), cohort and case–control studies were considered for the systematic review. Non-human studies, conference abstracts, case reports and non-English studies were excluded.

This search was completed on the PubMed and Ovid Medline databases on the 17th of October, 2022. The search criteria for PubMed were (sleep apnoea, obstructive[MeSH Terms]) AND (bariatric surgery[MeSH Terms]). The search criteria for Ovid Medline were Sleep Apnoea, Obstructive/AND Bariatric Surgery/. Duplicates were removed using the reference management software Endnote 20 [20]. The title and abstract of each article were assessed independently for their relevance to the research question. The full texts for suitable studies were retrieved and analysed to see if they met the eligibility criteria.

The Newcastle–Ottawa scale was used to assess the risk of bias for all cohort studies chosen for the systematic review [21]. This scale adopts a star-awarding system to assess the quality of non-randomised studies permitting the calculation of an overall quality score. The scale provided by this method has a maximum score of 10 points. 0–3 points indicate a high risk of bias, 4–6 points indicate a moderate risk and ≥ 7 points indicate a low risk of bias.

Summary data from all included studies included the year of publication, study design, setting, sample size, duration, follow-up period and key findings. Baseline characteristics of all participants included average age, gender, body mass index (BMI), smoking status and comorbidities such as hypertension (HTN) and depression.

The outcome variables included overall complications, cardiovascular complications, respiratory complications, ICU/HDU admission, 30-day readmission and length of stay (days). These outcome measures included dichotomous variables (complications, ICU/HDU admission and 30-day readmission) and continuous variables (length of stay).

All studies were added to RevMan 5.4 [22] and Open Meta-Analyst [23] and used to generate forest plots for each outcome measure. Risk ratio or relative risk was used as the effect measure for dichotomous variables. Mean difference was used as the effect measure for continuous variables. The *I*-squared (*I*^2^) and chi-squared statistics were used to assess the statistical heterogeneity of each outcome measure. The fixed-effects model was used if *I*^2^ was less than 50%, meaning the studies were relatively homogenous. If *I*^2^ was greater than 50%, the studies pooled were heterogenous and the random effects model was used [24]. The confidence interval was set at 95% and a *p*-value of less than 0.05 was considered significant for the effect measures calculated from the pooled studies.

## Results

The initial search returned 367 articles in PubMed and 255 articles in Ovid Medline which were then added to a reference manager software. Any duplicates found were then removed manually. This resulted in a total of 367 articles which met the search criteria. After this, the title and abstract of each article were assessed to see if the article or study would be relevant to the research question.

**Fig. 1:**
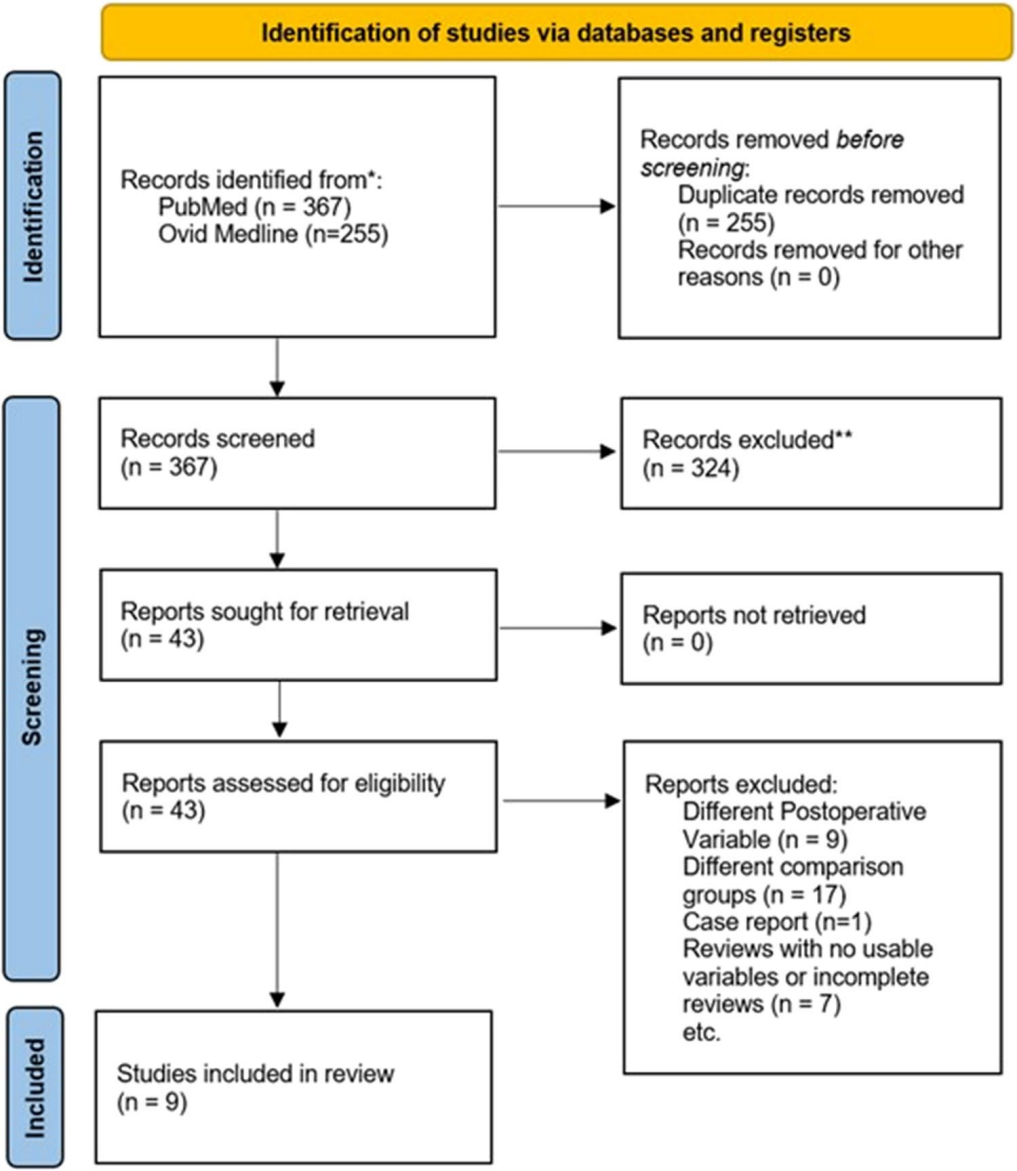
PRISMA 2020 flow diagram for systematic review showing the selection process of initial searches to the final number of included studies (Page et al., 2021)

This screening process resulted in 43 studies related to perioperative or postoperative outcomes for patients after bariatric surgery and had some mention of OSA patients (Fig. 1). These articles were then retrieved and analysed in detail to see if they were relevant to the systematic review. A few studies were removed from the selection because they were focused on different postoperative outcome measures. Case reports were also removed since they were not replicable and would not be suitable for this systematic review. At the end of this selection process, 9 studies were suitable for the systematic review and meta-analysis.

The majority of the studies selected for the systematic review were retrospective cohort studies. Two prospective cohort studies also satisfied the inclusion criteria. All patients who did not fit the criteria of OSA patients and non-OSA patients undergoing bariatric surgery were removed from consideration. A total of 5143 patients were included across all cohort studies used in the systematic review and meta-analysis. These patients were separated based on the presence of OSA. The average age of patients from 42.9 to 49.3 in the OSA group and from 39.4 to 46.0 in the non-OSA group. The gender distribution ranged from 14.4 to 72% male in the OSA group and 5.6 to 69.2% male in the non-OSA group. The largest cohort study included in the systematic review consisted of 1094 patients and the smallest cohort study included 277 patients. Follow-up duration was usually 30 days but one study followed patients over a longer period of around six years. (Table 1). Quality assessment of studies was provided in Table 2.

**Table 1:**
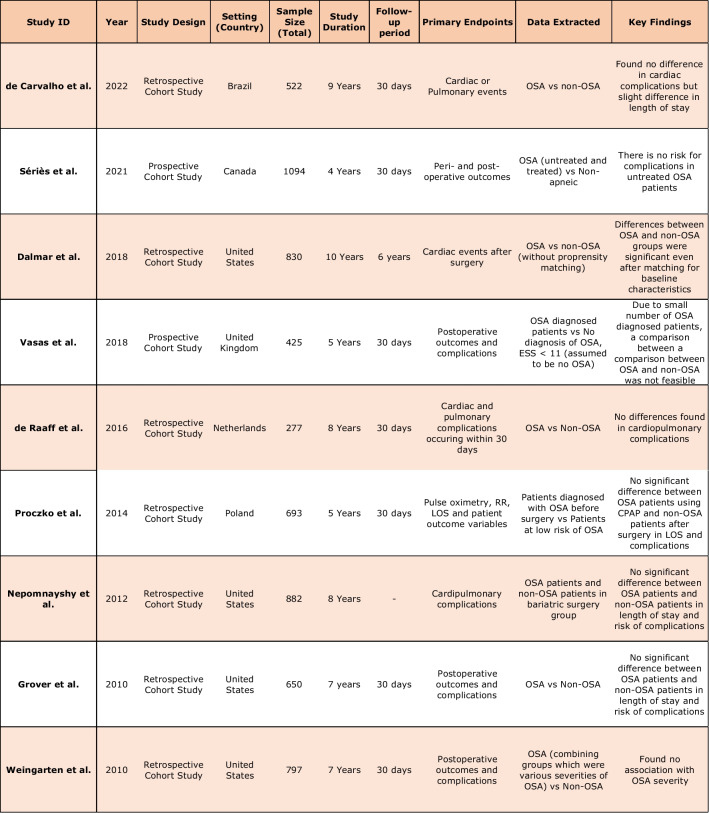

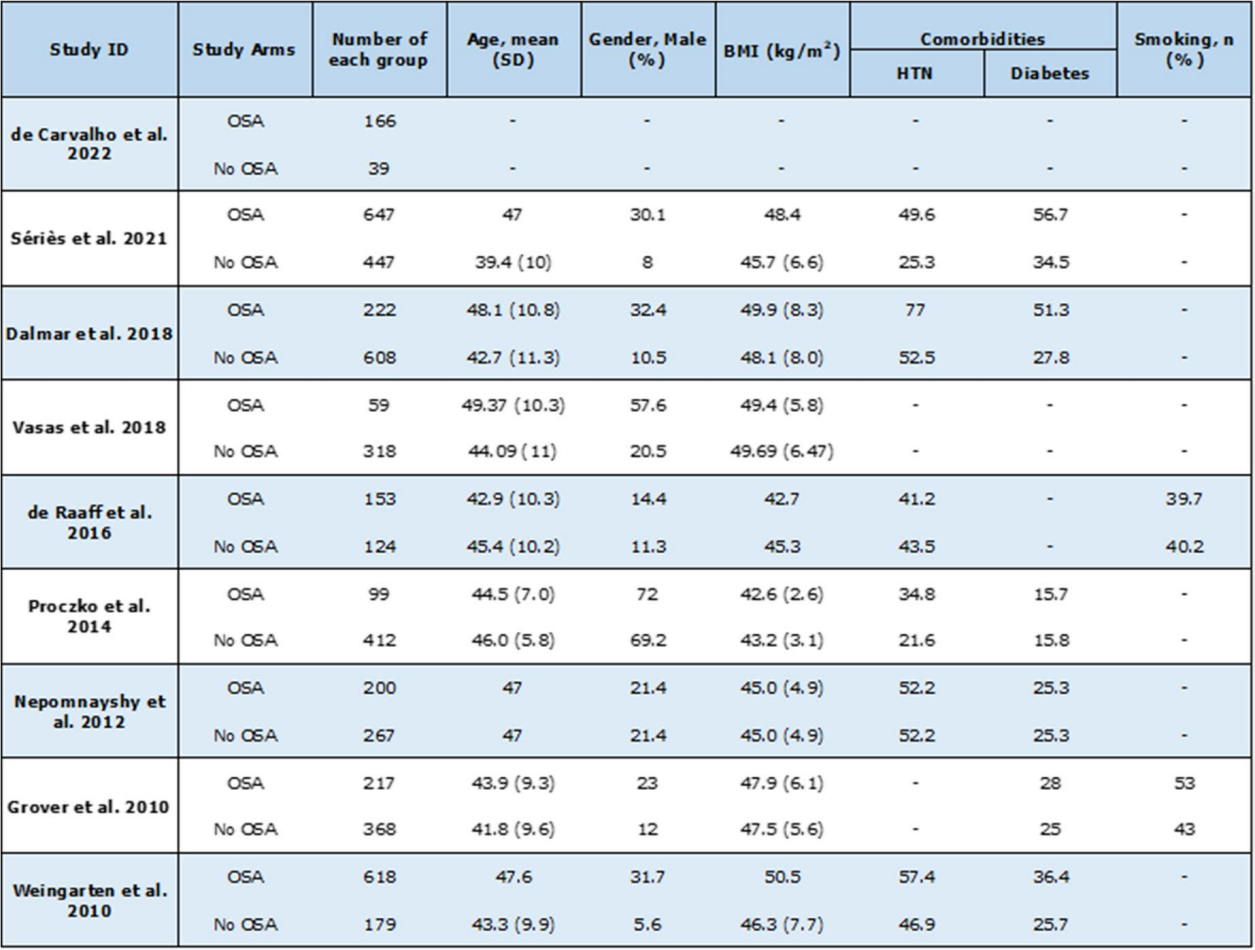
Summary of characteristics of included studies [25–33]

**Table 2:**
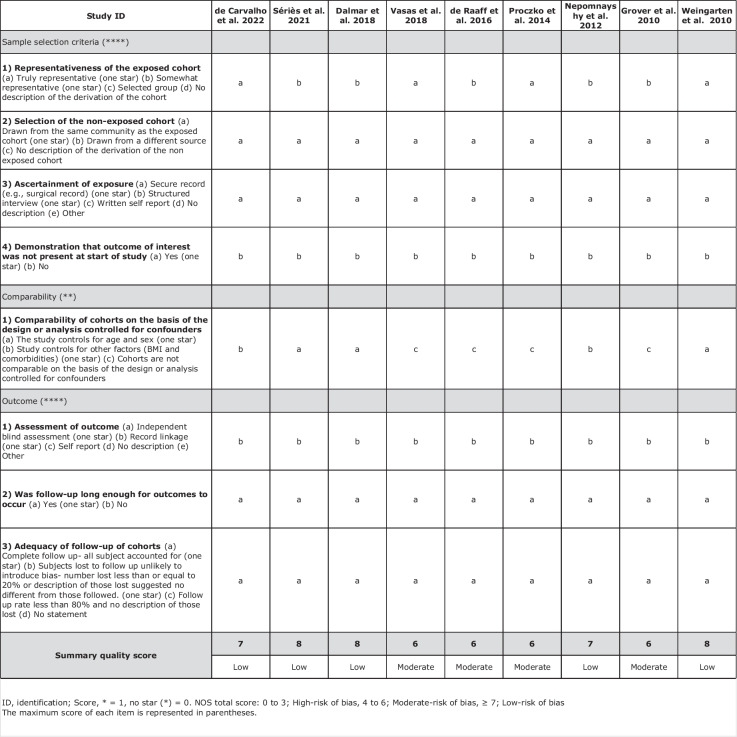
Quality assessment criteria used for cohort studies (Newcastle-Ottawa scale for cohort studies)

ID, identification; score, * = 1, no star (*) = 0. NOS total score: 0 to 3; high-risk of bias, 4 to 6; moderate-risk of bias, ≥ 7; low-risk of bias

The maximum score of each item is represented in parentheses

**Fig. 2:**
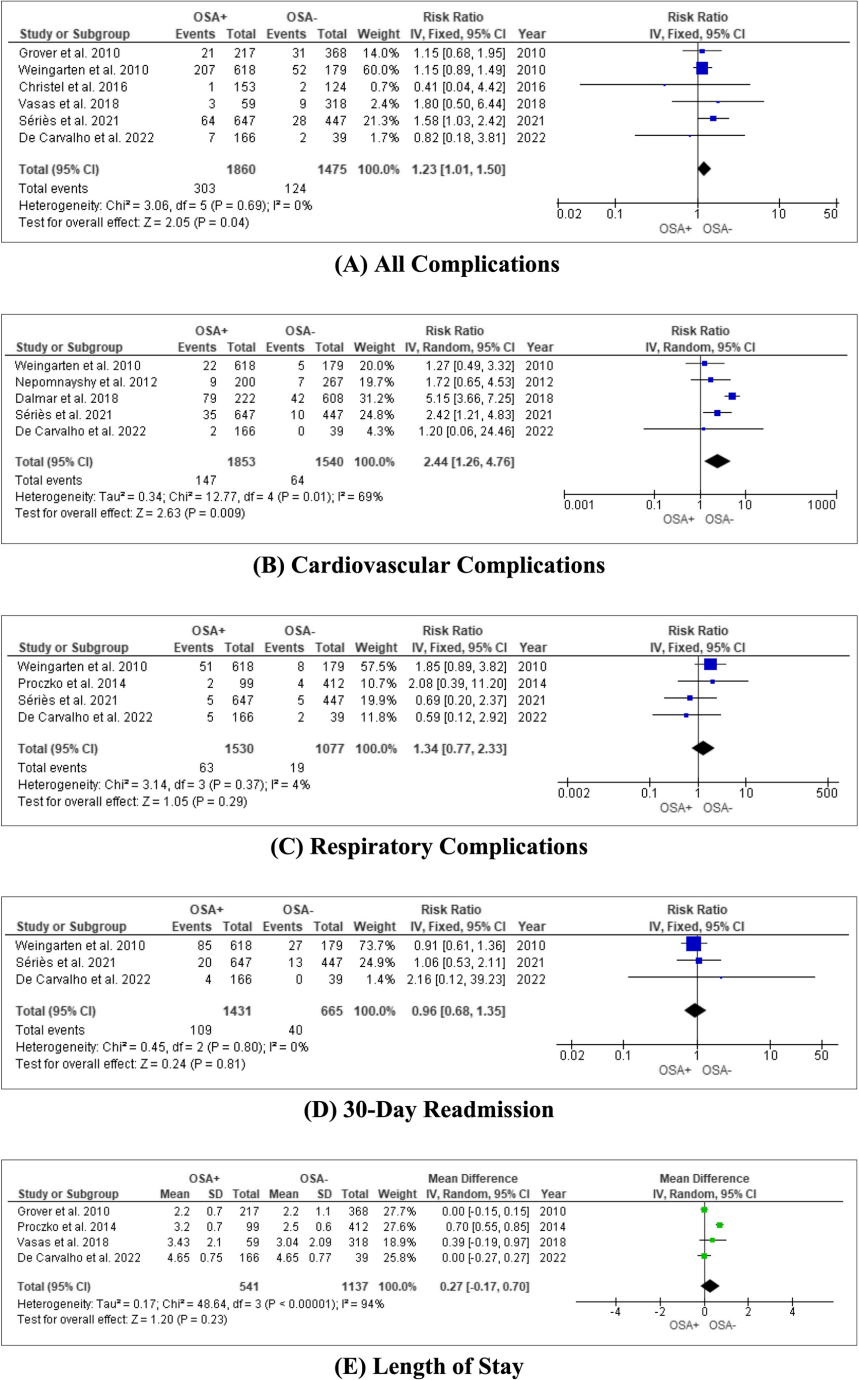
Forest plots comparing the pooled effect of studies comparing the peri- and postoperative outcomes for OSA and non-OSA patients undergoing bariatric surgery in terms of (**A**) All complications showing statistically significant increased risk in the OSA cohort compared to the non-OSA cohort. (**B**) Cardiovascular complications showing statistically significant increased risk in the OSA cohort compared to the non-OSA cohort. (**C**) Respiratory complications showing no significant difference between OSA and non-OSA cohorts. (**D**) 30-day readmission showing no significant difference between OSA and non-OSA cohorts. (**E**) Length of stay showing no significant difference between OSA and non-OSA cohorts

The pooled effect of the studies showed a statistically significant increase in the risk of overall complications (RR = 1.23, 95% CI: [1.01, 1.5], *P* = 0.04) between OSA patients and non-OSA patients, largely driven by the increased risk of cardiovascular complications (RR = 2.44, 95% CI: [1.26, 4.76], *P* = 0.009). Cardiovascular complications include acute coronary artery disease, heart failure including cor pulmonale, clinically relevant dysrhythmia (defined as atrial fibrillation/flutter, the presence of frequent ventricular ectopy on ECG, or implanted pacemaker). There was also no increased risk of respiratory complications (RR = 1.34, 95% CI: [0.77, 2.33], *P* = 0.29) between the two groups. All other variables under consideration showed no significantly increased risk between the two groups (Fig. 2).

## Discussion

Studies in the past have shown an increased risk in most of these outcome variables in the OSA cohort [17]. This appears to be less of an issue in the bariatric surgery population. Patients with OSA were more at risk of these outcomes when undergoing other types of surgery especially orthopaedic procedures [31]. However, preoperative and postoperative CPAP use is common during bariatric procedures and reduces the length of stay and risk of complications in both OSA and non-OSA patients [18]. CPAP use was in most of the studies used in the systematic review.

There are few systematic reviews based on the outcomes of OSA patients after bariatric surgery. One systematic review found no increased length of stay or risk of complications in patients with OSA [34]. The normalisation of CPAP use during bariatric surgery may be causing a lower risk of complications in the bariatric surgery population compared to patients undergoing other surgery types. This study also suggests that it may not be necessary to always admit patients with OSA undergoing bariatric surgery to the ICU. The importance of CPAP use preoperatively and postoperatively cannot be understated.

Compared to other countries in Europe, there is a relative shortage of hospital beds in the UK compared to its population [23]. Hospitals can better manage their resources by knowing which patient populations are more likely to require beds. Outcome measures such as the length of stay in the hospital and the need for ICU admission can help in this regard. From the data gathered in this review, it seems unlikely that a patient undergoing bariatric surgery will need to be managed differently depending on the presence of OSA. However, it must be noted that patients with OSA are at higher risk of complications (especially cardiovascular).

Unfortunately, it is difficult to make any conclusions at this stage. There is far too little literature on the research question. In addition, no studies included in the systematic review and meta-analysis were randomised control trials. The reliability of cohort studies is highly dependent on the research protocol. It seemed that the outcome measures for these studies were chosen before data collection. As such, there may have been some selective reporting of outcomes.

There was also a great deal of heterogeneity between the studies in terms of cohorts and findings. This is seen especially in the cardiovascular complications outcome. It may be the case that the higher weight of certain studies led to an exaggerated risk ratio. In addition to this, one study purely focused on cardiovascular complications for a longer follow-up period [27]. This study however found that the increased risk of cardiovascular complications in the OSA group stays even after matching for baseline characteristics.

Some assumptions were made when collecting data and grouping it into the generalised study arms: OSA and non-OSA. For example, it is not possible to completely rule out OSA using the Stop-Bang questionnaire and Epworth sleepiness score. However, this assumption was also made by the studies used for the meta-analysis [28, 30]. Therefore, the conclusion provided by these results may be misleading.

This systematic review and meta-analysis show that patients with OSA are at increased risk of cardiac complications following bariatric surgery compared to patients without OSA. There is no increased risk of respiratory complications between the two groups. There is also no difference in length of stay, ICU admission or 30-day readmission between the two groups.

## Data Availability

Data will be provided following a reasonable request.
